# Pharmacokinetic Characterization to Enable Medicine Use in Pregnancy, the Potential Role of Physiologically‐Based Pharmacokinetic Modeling: A Regulatory Perspective

**DOI:** 10.1002/psp4.12551

**Published:** 2020-08-27

**Authors:** Susan Cole, Paola Coppola, Essam Kerwash, Janet Nooney, Siu Ping Lam

**Affiliations:** ^1^ Medicines and Healthcare Products Regulatory Agency London UK

It is generally recommended that use of medicines should be avoided during pregnancy; however, sometimes the use of a medicine is essential to safeguard the health of the mother and baby. A limited number of medicines are, however, specifically licensed for use in pregnancy.

This article considers the potential of physiologically‐based pharmacokinetic (PBPK) modeling as a predictive tool to investigate the expected systemic exposure of medicines in pregnant women and to support possible dosing adjustment to maintain efficacy.

## Use of Medicines During Pregnancy

Self‐reported medicine use by pregnant women surveyed in Europe, the Americas, and Australia in 2011 revealed that more than 80% of women used at least one medicinal product during pregnancy. Some regional differences existed; however, 67% of women self‐reported use of nonprescription or over‐the‐counter medicines, whereas 17% of women reported medicine use for chronic conditions.^1^

Treatments are often essential for pregnant women with underlying conditions where poor maternal health can itself pose risks for the pregnancy, for example, depression, epilepsy, or cardiovascular conditions. In addition, treatment with antiviral agents are required, e.g., for HIV patients to prevent transmission of the virus or for malaria treatment. Other medicines may be required for conditions such as pre‐eclampsia or diabetes that may develop during pregnancy. There is, however, limited scientific information to support the safe and optimal use of medicines in pregnancy. Only five prescription medicines (i.e., amoxicillin, labetalol, diazoxidine injection, doxylamine succinate/pyridoxine hydrochloride, sodium feredetate) are specifically licensed in the United Kingdom for nonobstetric use in pregnancy.

The necessity to treat the mother needs to be balanced against potential adverse effects on the fetus. The benefit‐risk decision to inform medicine use in pregnancy relies on the available information about the effect of the medicine on pregnancy outcomes and the effect of pregnancy on the medicine. Changes in drug systemic exposure during pregnancy could increase risks for mother and baby. Particularly, underexposure may lead to loss of efficacy and reduced control of maternal health with associated risks for fetal development, whereas overexposure could result in exposing the mother and the fetus to unnecessarily high doses, increasing the risk of adverse effects. Optimal formulations and dosages are not usually investigated in pregnant women, and for most medications it is unknown whether a single dosing regimen will be sufficient throughout pregnancy or whether one or several changes would be required as pregnancy advances.

At the time of licensing, most new medicines in the European Union advise avoiding use in pregnancy because of a lack of sufficient studies in pregnant women. The European Medicines Agency and US Food and Drug Administration recommend that marketing authorization applicants should perform, where possible, pharmacokinetic (PK) and pharmacodynamic studies in pregnant women to understand better how pregnancy affects the blood levels of medicines commonly used, particularly during the first trimester, and to develop evidence‐based dosing and frequency of administration guidance for use in pregnancy.^2^, ^3^ However, concerns about taking part in clinical trials because of unknown benefit‐risk profiles of investigational medicines in both the mother and fetus may lead to difficulties in recruiting pregnant women, which in turn limits the generation of data.

The resulting lack of information on safety and appropriate doses leads to health care professionals being reluctant to prescribe newer medicines during pregnancy, which may be more efficacious and/or have a better general safety profile than older medicines.

## The Importance of PK Characterization of Medicine in Pregnant Women

It is known that considerable physiological changes occur during all gestational trimesters and that these may alter the way the body normally deals with administered medicines, consequently, the PK and/or pharmacodynamics of medicines may change. As pregnancy advances, clinically important alterations in the absorption, distribution, metabolism, and excretion of medicines can be observed.

Nausea, vomiting, and alterations in gastric pH and gastrointestinal tract motility may alter the absorption process, which, conversely, may be enhanced by the increased cardiac output and intestinal blood flow. The central volume of distribution increases during pregnancy because of a plasma volume increase of up to ~ 42% by the third trimester, and an increase in blood flow to the uterus will mean increased delivery of lipophilic medicines to the placenta and the fetus. Metabolizing enzymes (e.g., cytochrome P450) may have increased, or decreased, activity during pregnancy leading to altered drug clearance. The renal glomerular filtration rate also increases throughout pregnancy and can lead to higher clearance of renally excreted medicines (e.g., lithium, digoxin). The expression and activities of transporters, which are extensively expressed in several organs, including the placenta–fetus compartment, are also expected; however, limited data are currently available in this area.^4^

## A Role for PBPK Modeling?

PBPK modeling is increasingly used in drug development and regulatory submissions, and the purpose of the models has expanded from the assessment of drug interactions to the assessment of PK in special populations.^5^ PBPK modeling uses a mathematical approach to develop an understanding of how drug systemic exposure can be influenced by changes in physiology. Several papers have been published on the use of PBPK modeling to describe anatomical and physiological changes during pregnancy, and these models have been evaluated with different test drugs eliminated via filtration, transport, or metabolism.^6^, ^7^, ^8^ Most models relate to the late second/third gestational trimester, and an extensive understanding of the impact of physiological changes throughout pregnancy on medicine PK and pharmacodynamics is yet to be completed. PBPK methodology could be used as a predictive tool to support a benefit‐risk decision and for selection of the adequate dose in pregnancy.

In the US FDA's draft guidance,^3^ modeling and simulations, including PBPK, are suggested to support the design of clinical PK studies in pregnant women. It is apparent that modeling could also be used to identify which medicines are more likely to be affected by pregnancy and, therefore, would be a priority to obtain clinical data in pregnant women. Eventually, there may be situations where the confidence in the PBPK model is such that it can be used to support extrapolation of efficacy and safety data from healthy volunteers to pregnant women without any clinical data.

Dosing based solely on PK, whether measured or predicted, is considered an extrapolation in EU regulatory terms, and a framework has recently been published for children.^9^ This framework could be usefully applied to pregnancy. A comprehensive PBPK pregnancy model framework could bridge the gaps in data to support a prospective investigation of the exposure–response relationship, which is essential for predicting necessary dose changes to maintain maternal health.

## Medicines and Healthcare Products Regulatory Agency Project on PBPK Modeling for Drug Use in Pregnancy

The Medicines and Healthcare Products Regulatory Agency (MHRA) is collaborating with the Bill and Melinda Gates Foundation in Seattle, WA, to evaluate existing PBPK models to inform dosing in pregnant women in the United Kingdom.

The project aims to generate new insights on drug disposition during pregnancy that can have an impact on the health of pregnant women and give obstetricians further clarity on optimal doses of medicines in pregnant women, when such treatments are considered necessary. It also aims to increase clinician's awareness in the use of predictive tools such as PBPK models to support more informed dosing in pregnancy.

The project will facilitate the provision of quantitative PK data to support dose selection for medicines of interest and to fill gaps in the knowledge to inform an improved benefit‐risk assessment of the medicines in pregnancy. The focus of this work is to use PBPK modeling to use the available PK data to understand existing system components concerning pregnancy in PBPK models (virtual pregnant population model). The initial phase of the project will focus on maternal exposure. It is intended that future work would focus on exposure of the fetus. This will assist in the understanding of passage of drugs across the placenta and as a minimum should be useful to highlight those drugs for which exposure to the fetus would not be predicted.

The MHRA has identified a list of medicines commonly used in pregnancy for which the impact of pregnancy on their PK will be investigated using currently available information and PBPK modeling. This list includes approximately 200 medicines in several therapeutic areas, including diabetes, epilepsy, antidepressants, antibiotics, antihypertensives, pain killers, corticosteroids, antivirals, immunosuppressants, anesthetics, and antihistamines. Clinical PK data in pregnancy will be collected from the literature, clinical trial databases, or data held in‐house from historical regulatory submissions and used to qualify/validate the available PBPK models.

Depending on an understanding of the uncertainties in the models, confidence in them to support regulatory decisions can be addressed. Drug systemic exposures predicted in a virtual pregnant population can, as a minimum, support optimal design of any planned PK trials and could inform the information given to trial participants on the risks and benefits of investigational medicines. High confidence in the models would be required, however, for high‐impact applications,^10^ such as to inform doses in pregnancy without any clinical data in this population.

The MHRA will also work with healthcare professionals. Throughout the project, training sessions and workshops will be organized to introduce PK and PBPK to obstetricians and physicians. Findings from the PBPK modeling will be used as case examples to facilitate physicians’ understanding of quantitative methods and conversely to receive feedback from physicians on further improving predictability of PBPK models.

## Conclusion

Clinical studies in pregnant women are required to inform and ensure the safe and effective use of medicines in this population. However, most clinical trials normally exclude pregnant women based on possible risks to the fetus. PBPK models can provide a conceptual understanding and useful quantitative data on PK. This can be used to support benefit‐risk assessment and dose selection or to indicate medicines where further clinical data are required during different stages of pregnancy, especially where there is currently a sparsity of data. The confidence in these models needs to be assessed before their full utility, e.g., high‐impact uses in regulatory applications and for potential use by clinicians in the field, can be concluded. Further details and results will be made publicly available in the future.

## Funding

This project received a grant from the Bill & Melinda Gates Foundation, Seattle, WA, USA.

## Conflict of Interest

All authors declared no competing interests for this work.

## Disclaimer

The views expressed in this article are the personal views of the authors and may not be understood or quoted as being made on behalf of or reflecting the position of the regulatory agency or other organizations with which the authors are affiliated.
